# Multiple brain abscesses treated by extraction of the maxillary molars with chronic apical lesion to remove the source of infection

**DOI:** 10.1186/s40902-019-0208-2

**Published:** 2019-07-03

**Authors:** Ki-Hyun Jung, Seong-Su Ro, Seong-Won Lee, Jae-Yoon Jeon, Chang-Joo Park, Kyung-Gyun Hwang

**Affiliations:** Department of Dentistry/Oral and Maxillofacial Surgery, College of Medicine, Hanyang University, 222 Wangsimni-ro, Seongdong-gu, Seoul, 04763 South Korea

**Keywords:** Brain abscess, Odontogenic infection, Extraction

## Abstract

**Background:**

Brain abscess is a life-threatening condition that occurs due to complications during a neurosurgical procedure, direct cranial trauma, or the presence of local or distal infection. Infection in the oral cavity can also be considered a source of brain abscess.

**Case presentation:**

A 45-year-old male patient was transported with brain abscess in the subcortical white matter. Navigation-guided abscess aspiration and drainage was performed in the right mid-frontal lobe, but the symptoms continued to worsen after the procedure. A panoramic radiograph showed alveolar bone resorption around the maxillary molars. The compromised maxillary molars were extracted under local anesthesia, and antibiotics were applied based on findings from bacterial culture. A brain MRI confirmed that the three brain abscesses in the frontal lobe were reduced in size, and the patient’s symptoms began to improve after the extractions.

**Conclusion:**

This is a rare case report about multiple uncontrolled brain abscesses treated by removal of infection through the extraction of maxillary molars with odontogenic infection. Untreated odontogenic infection can also be considered a cause of brain abscess. Therefore, it is necessary to recognize the possibility that untreated odontogenic infection can lead to serious systemic inflammatory diseases such as brain abscess. Through a multidisciplinary approach to diagnosis and treatment, physicians should be encouraged to consider odontogenic infections as a potential cause of brain abscesses.

## Background

A brain abscess is a serious and life-threatening condition that requires prompt neurosurgical management. Brain abscesses can be due to complications during a neurosurgical procedure, direct cranial trauma, and local or distal infection [1]. The most common causes of brain abscess are local infection including peri-cerebral infections that spread to the brain along the craniofacial area, hematogenous infections that spread into the cranial cavity through arteries or veins, and direct infections that spread into brain tissues when bacteria enter through a head and neck area. A direct infection usually originates from an odontogenic infection [2]. However, other infections, such as maxillary sinusitis, meningitis, or suppurative cellulitis, can flow along the fascia, arrive in the cranial cavity, and spread through the blood vessels. Odontogenic infections are a hematogenous cause of brain abscesses, but are only responsible for fewer than 2% of cases [3]. Because of the low incidence of brain abscesses with a dental origin, clinicians can have difficulty reaching a correct diagnosis, which can result in delayed treatment. Despite the development of improved image modalities and diagnostic technology, it is still challenging for clinicians to reach a proper diagnosis when brain abscesses are caused by odontogenic infection.

This case report presents a unique case of multiple brain abscesses induced by an odontogenic infection that remained uncontrolled by a general neurosurgical procedure and aggressive antibiotic treatment, however, was successfully treated by extracting maxillary molar that exhibited chronic apical lesion.

## Case presentation

A 45-year-old male patient visited the emergency room in XXX University Hospital with right facial spasms, tingling and twisting of the right arm, paresthesia, and dysarthria. The patient had no medical history or underlying disease, with the exception of being hospitalized for pneumonia 1 month previously. The patient consumed alcohol occasionally and had smoked 1.5 packs of cigarettes per day for 25 years. When admitted to the ER, the patient was conscious with a blood pressure of 150/88 mmHg, heart rate of 77 beats/min, respiratory rate of 20 breaths/min, and body temperature of 37.0 °C. According to the laboratory tests on a peripheral blood sample, the white blood cell count was 13,900/mm^3^ (86% neutrophil), hemoglobin concentration was 13.5 g/dl, and platelet count was 248,000/mm^3^. Biochemical tests showed a blood urea nitrogen concentration of 11 mg/dl, creatinine concentration of 0.6 mg/dl, C-reactive protein concentration of 0.63 mg/dl, blood glucose of 140 mg/dl, and HbA1c of 5.5%. No bacterial growth was observed in cerebrospinal fluid culture, and electroencephalography (EEG) indicated normal patterns. On the day he was admitted to the ER (day 1), brain CT scan revealed three low-density oval lesions (13 mm, 9 mm, and 15 mm) in the right mid-frontal region and in the left and right high-frontal subcortical white matter. According to MRI on the second day (day 2), the oval lesions with diffusion restriction in the same areas appeared swollen, and blood volume and flow in the perilesional areas were decreased. Empirical antibiotic treatment was initiated as metronidazole 500 mg every 8 h, cefotaxime 2 g every 12 h, and dexamethasone 5 mg every 6 h administered through intravenous infusion.

**Fig. 1:**
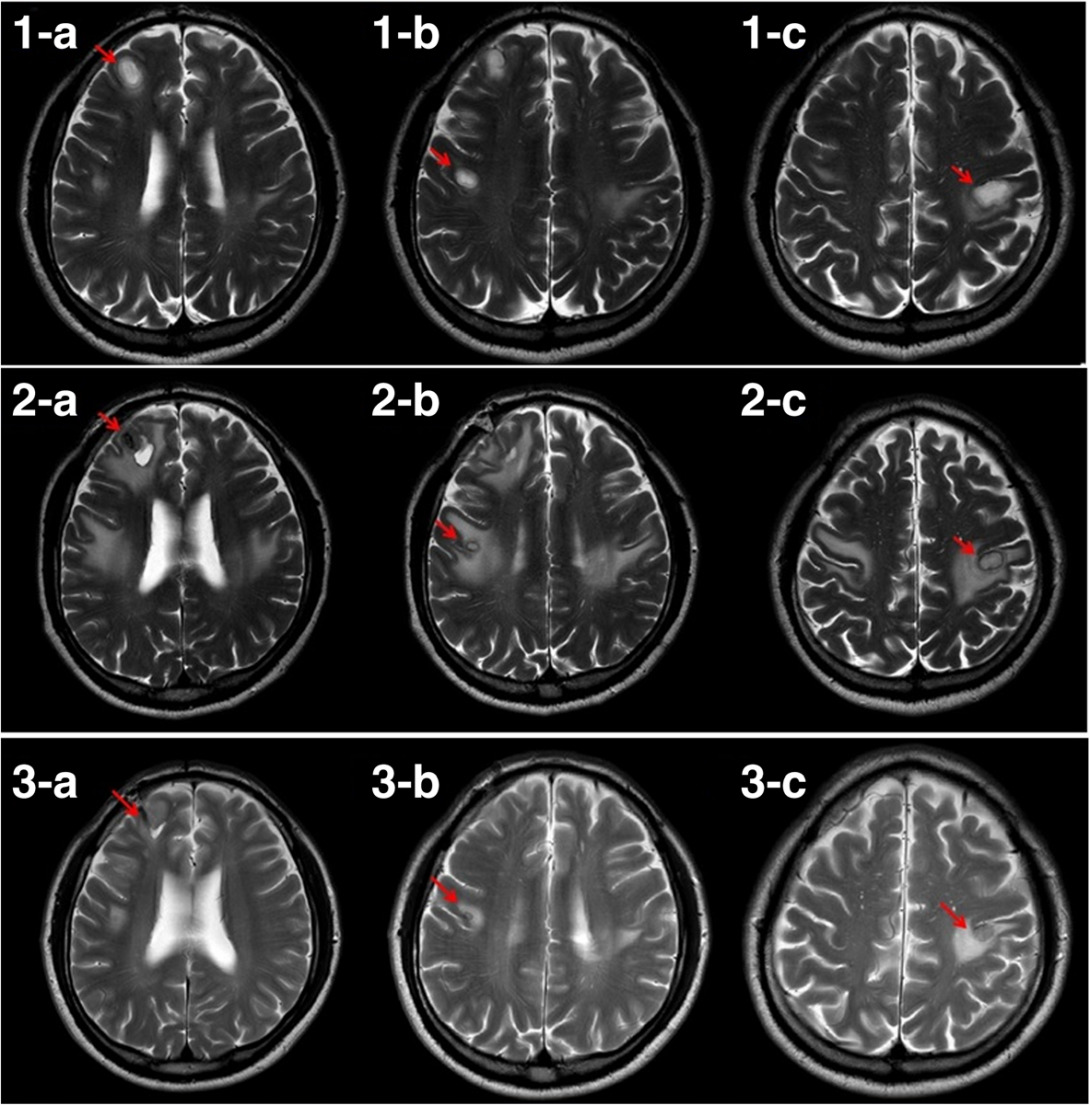
Magnetic resonance images of a 45-year-old male admitted with right facial spasm, tingling and twisting of the right arm, paresthesia, and dysarthria. A high T2-weighted signal was observed in the right mid-frontal region and in the left and right high-frontal subcortical white matter (WM). Top: three regions were well-defined, approximately 13 mm, 9 mm, and 15 mm low-density ovoid lesions, with diffusion restriction and perilesional edema. Middle: the post-operative state; abscess drainage of the right mid-frontal lobe area. The size of several ovoid lesions decreased in both the left and right high-frontal subcortical WM. Bottom: after extraction of the periodontally involved teeth, the size of the lesions in the right mid-frontal and both the left and right high-frontal subcortical WM decreased significantly. A, right mid-frontal; B, right high-frontal; C, left high-frontal subcortical area. Red arrows identify the three brain abscesses

Following a consultation with a representative from the Department of Infectious Disease the following day, cefotaxime 2 g was replaced with ceftriaxone 2 g, dexamethasone 5 mg was continued, and metronidazole 500 mg was discontinued. On day 12 of hospitalization, a follow-up MRI scan showed that the size of the lesions had not changed, and the boundaries of the lesions were more defined with diminished edema (Fig. 1). The images from a follow-up CT scan on day 20 confirmed a clear boundary of peripheral edema, and the patient was transferred to the Department of Neurosurgery for surgical drainage. On the following day (day 21), navigation-guided abscess aspiration and drainage was performed in the right mid-frontal lobe under general anesthesia. Abscess fluid was collected during the operation and then incubated in aerobic and anaerobic conditions, followed by Gram staining. After surgery, the patient was transferred to the Department of Neurology, and ceftriaxone 2 g every 12 h and metronidazole 500 mg every 8 h were administered through intravenous infusion. The patient’s symptoms continued to worsen after the operation, even though ceftriaxone 2 g every 12 h and metronidazole 500 mg every 8 h were continued and vancomycin 1 g every 8 h was added. On day 34, the patient was transferred to the Department of Infectious Diseases to continue with this aggressive antibiotic treatment (Table 1). The patient’s symptoms improved very slowly. It was suspected, therefore, that there was another source of infection, and an intraoral lesion was detected.

**Table 1:** Antibiotic regimen

Days 1–5	Days 6–20	Days 21–33	Days 34–44	Days 45–60
Cefotaxime 2 g IV q12hr Metronidazole 500 mg IV q8hr Dexamethasone 5 mg IV q6hr	Ceftriaxone 2 g IV q12hr Dexamethasone 5 mg IV q6hr	Ceftriaxone 2 g IV q12hr Metronidazole 500 mg IV q8hr	Ceftriaxone 2 g IV q12hr Metronidazole 500 mg IV q8hr Vancomycin 1 g IV q8hr	Augmentin 2.4 g IV q8hr

*IV, intravenous*

**Fig. 2:**
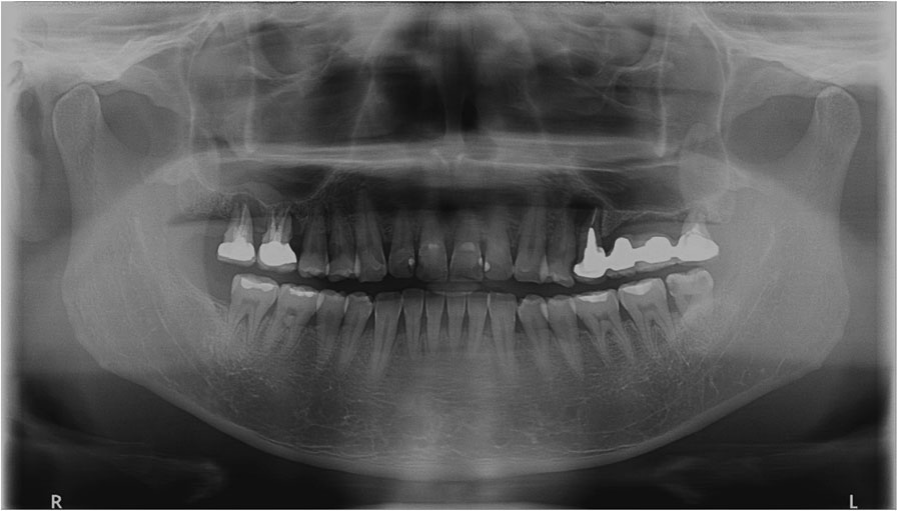
Panoramic radiograph: vertical alveolar bone loss and periapical abscesses were observed in the right maxillary first and second molars, the left maxillary second premolar, and the third molar. The sites were treated with root canal therapy

**Fig. 3:**
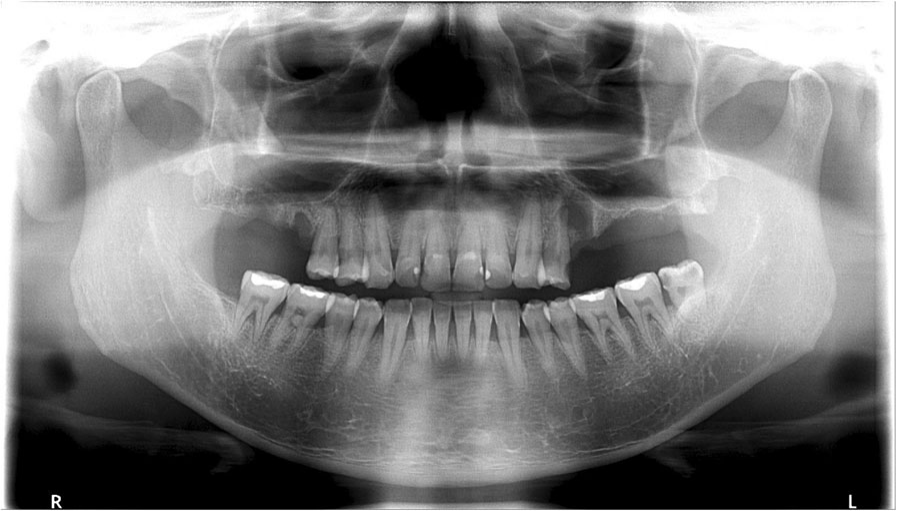
Panoramic view after extraction of the right maxillary first and second molars, the left maxillary second premolar, and the left maxillary third molar

The patient was sent to the Department of Oral and Maxillofacial Surgery in XXX University Hospital on day 41 for consultation. A panoramic X-ray revealed radiolucency around the right maxillary first molar, right maxillary second molar, left maxillary first premolar, and left maxillary third molar. The patient was diagnosed with chronic periodontitis and periapical abscesses (Fig. 2). On day 44, the right maxillary first molar, maxillary second molar, left maxillary first premolar, and left maxillary third molar were extracted under local anesthesia in the Department of Oral and Maxillofacial Surgery (Fig. 3). Abscess fluid from the tooth extraction socket was incubated in aerobic and anaerobic conditions, followed by Gram staining; *Streptococcus anginosus* was identified. Antibiotic treatment was changed to IV infusion of amoxicillin/clavulanic acid 2.4 g every 8 h. The patient’s symptoms started to improve on day 47 (Table 2). On day 58, brain MRI images confirmed that the three brain abscesses in the frontal lobe were reduced in size, and perilesional edema was confirmed as the interval resolving state (Fig. 1, bottom). The patient was prescribed amoxicillin/clavulanic acid 625 mg every 8 h by oral administration and discharged from the hospital on day 61.

**Table 2:** Symptoms during hospitalization

Onset	NR (Guri)	NS (Guri)	NR (Guri)	ID (Guri)	ID (Seoul)	Discharge
	Day 1	Day 20	Day 24 (3 days after surgical drainage)	Day 34	Day 47 (3 days after extraction of teeth)	Day 61
Right facial spasm	+	–	–	–	–	–
Rt. U/Ext weakness	+	+	+	+	–	–
Rt. U/Ext Tingling sensation	+	+	+	+	–	–
Dysarthria	+	+	+	+	+	+

*Rt. U/Ext; right-hand upper-extremity*

## Discussion and conclusion

Brain abscesses tend to occur in men younger than 60 years of age (they are uncommon in children), and the main areas affected are the temporal lobe (42%) and cerebellum (30%) [1, 2]. The 40% of brain abscesses are caused by chronic otitis media or mastoiditis, 10% by maxillary sinusitis or paranasal sinusitis, and 50% through the spread of cardiac or pulmonary infections. In rare cases, brain abscesses can occur after dental treatment or cranial trauma. Therefore, brain abscesses are associated with metastatic foci of infections such as cardiac or pulmonary infections, which need to be evaluated and treated in the early stage of disease course, as well as contiguous foci of infection such as otitis, mastoiditis, and sinusitis for avoiding severe complications [2–4]. Metastatic infections mediated by microbes from other parts of the body are transferred through the circulatory system or anatomical fascia. Microorganisms are not limited to the periodontal area. Microorganisms gain access to the blood or circulatory system, and the bacteria can migrate to adjacent tissues or other distant organs to cause infection. When bacteria arrive in the brain, localized encephalitis is established, and white blood cells and inflammatory exudate accumulate, causing infective thrombosis [5]. When conventional antibiotic regimens do not control the infection, as in the present case, diagnosis and examination of persistent bacterial inflow sources are necessary. Antibiotic coverage or removal of infection is not effective in controlling inflammation in the parenchyma region. As seen in this case, simultaneous examination and treatment of the distant site can effectively control inflammation in the parenchyma region.

To diagnose a brain abscess, diagnostic imaging, such as a cephalogram, CT, MRI, or angiography, should be used in addition to a clinical examination to distinguish from a brain tumor or hematoma. The use of CT scanning has provided early diagnosis, exact site, characterization, and staging of the abscess [6]. In addition, MRI can be used for the accurate diagnosis of brain abscesses and for detecting the precise location of abscesses because of its ability to distinguish between brain edema and normal brain tissue and its increased sensitivity compared to CTs for detection of early cerebritis. Lesions in the brain may not be localized and may originate from other parts of the body. Therefore, if a remote infection is suspected, imaging multiple parts of the body with chest radiography and a head and neck evaluation may be necessary. Intra-oral infection is a rare cause of brain abscess, and dental radiography is necessary for proper diagnosis. It is difficult to accurately diagnose odontogenic disease by dental radiography alone because of acute inflammatory progression, which is a chronic disease, and inflammation of the soft tissue around the alveolar bone and formation of the abscess. In the case of cerebral abscess, which does not respond to long-term treatment, a thorough oral clinical examination can be performed concurrently with a radiologic examination to determine the exact cause or it would be better to eliminate the potentially causal odontogenic foci for improvement of oral hygiene [7]. However, further studies about the decision criteria for oral and maxillofacial surgeons to eliminate suspected casual teeth will be necessary.

The brain abscess was caused by poly-micro-organism, and it is difficult to identify accurate pathogens. For brain abscess that does not respond to long-term use of antibiotics or surgical treatment, it is necessary to assess the infection focus of other body parts. In brain abscess with a suspected remote infection, it is reasonable to evaluate the head and neck areas close to the brain carefully and chronic infectious diseases such as odontogenic infection can cause such pathology. It has been reported that the *Streptococcus anginosus* group (SAG) is sensitive to penicillin, ampicillin, cephalosporin, clindamycin, and erythromycin, no matter the exact species of the bacterium. Recently, however, the appearance of antibiotic-resistant bacteria, especially penicillin-resistant bacteria, is common; for this reason, careful selection of antibiotics is required [8]. In this case, the *S. anginosus* identified in the abscess fluid from the tooth extraction socket had a tolerance to penicillin, and the bacteria were eliminated with ceftriaxone during the first 20 days of hospitalization. Ceftriaxone and metronidazole administered from day 21 and vancomycin administered from day 34 should have repressed the growth of bacteria even further. However, the symptoms worsened, and it appeared to be because the source of the infection was not removed. Table 1 shows the antibiotic administration during the patient’s hospitalization. The primary treatment goal is to control the infection through the use of antibiotics. However, bacteria that have resistance to frequently used antibiotics have been reported, and bacterial pathogens of periodontal disease and systemic spread could not be controlled with conventional antibiotics. In this case, bacterial cultures were performed for the brain abscess to prescribe antibiotics, but there was no response to the antibiotics or surgical treatment. It indicates that the bacterial culture performed in the abscess around the periodontal tissue can be informative, and the efficacy of the antibiotic prescription was observed according to the results of bacterial culture at the time of extraction. Bacterial culture was performed in periodontal tissue at the same time as tooth extraction, and the brain abscess was effectively controlled using antibiotics as a result of bacterial culture. In conclusion, brain abscesses resulting from the odontogenic origin are rare; however, a multidisciplinary approach to diagnosis and treatment is important to consider the odontogenic infection as a potential cause of brain abscesses.

## Data Availability

Not applicable.

## Abbreviations

CT: Computer tomography
EEG: Electroencephalograph
ER: Emergency room
MRI: Magnetic resonance imaging
