# Correction to: Multiple brain abscesses treated by extraction of the maxillary molars with chronic apical lesion to remove the source of infection

**DOI:** 10.1186/s40902-019-0213-5

**Published:** 2019-07-22

**Authors:** Ki-Hyun Jung, Seong-Su Ro, Seong-Won Lee, Jae-Yoon Jeon, Chang-Joo Park, Kyung-Gyun Hwang

**Affiliations:** Department of Dentistry/Oral and Maxillofacial Surgery, College of Medicine, Hanyang University, 222 Wangsimni-ro, Seongdong-gu, Seoul 04763 South Korea

**Correction to: Maxillofac Plast Reconstr Surg (2019) 41:25**

**https://doi.org/10.1186/s40902-019-0208-2**

In the original publication of this article [1], the author wanted to correct the ‘XXX University Hospital’ in below two sentences. The ‘XXX University Hospital’ should be the ‘Hanyang University Hospital’.

1. ‘A 45-year-old male patient visited the emergency room in XXX University Hospital with right facial spasms, tingling and twisting of the right arm, paresthesia, and dysarthria. The original publication has been corrected. ’
2. ‘The patient was sent to the Department of Oral and Maxillofacial Surgery in XXX University Hospital on day 41 for consultation.’
